# Prognostic and Immunological Implications of FAM72A in Pan-Cancer and Functional Validations

**DOI:** 10.3390/ijms24010375

**Published:** 2022-12-26

**Authors:** Yuwen Bai, Kui Cao, Ping Zhang, Jianqun Ma, Jinhong Zhu

**Affiliations:** 1 Department of Thoracic Surgery, Harbin Medical University Cancer Hospital, 150 Haping Road, Harbin 150040, China; 2 Department of Clinical Laboratory, Biobank, Harbin Medical University Cancer Hospital, 150 Haping Road, Harbin 150040, China

**Keywords:** pan-cancer, FAM72A, prognosis, tumor microenvironment, LUAD

## Abstract

The family with sequence similarity 72 Member A (FAM72A) is overexpressed in several types of cancer. However, its contributions to tumorigenesis remain largely unknown. Based on The Cancer Genome Atlas (TCGA) database, FAM72A was upregulated across 33 types of cancer. Accordingly, high levels of FAM72A predicted inferior outcomes in half of the cancer types using survival analysis (the Kaplan-Meier curve and univariate Cox regression model). Receiver operating characteristic (ROC) analysis demonstrated that FAM72A showed high accuracy in distinguishing cancerous tissues from normal ones. FAM72A was correlated with immune and stromal scores and immune cell infiltrations in various tumors. Moreover, FAM72A was also associated with tumor mutation burden (TMB), microsatellite instability (MSI), and immune checkpoint genes. Immunophenoscore (IPS) further validated that the FAM72A^low^ tumor showed high immunogenicity and tended to respond to anti-PD1/PDL1/PDL2, anti-CTLA4 treatment, and combined immunotherapies. We also investigated the functional role of FAM72A in lung adenocarcinoma (LUAD). In vitro studies demonstrated that the ectopic expression of FAM72A accelerated the proliferation and migration of NSCLC cells, whereas silencing FAM72A showed the opposite effects on them. In short, FAM72A had prognostic potential and correlated with tumor immunogenicity in various tumors. Functional analysis indicated that FAM72A is an oncogene in LUAD.

## 1. Introduction

The family with sequence similarity 72 Member A (FAM72A), also known as Ugene, was initially identified in malignant colon cancers and overexpressed in several other common cancers, including breast cancer, lung cancer, uterus, and ovary cancer [1]. Accumulating evidence has indicated the critical role of FAM72A in tumorigenesis, but its functions are largely uncharacterized.

Several studies indicated that FAM72A functions by interacting with uracil DNA-glycosylase 2 (UNG2), a crucial enzyme in base excision repair (BER), catalyzing the replacement of deoxyuracil (dU) with deoxycytidine (dC) to avoid mutations [1,2,3,4]. Interestingly, two papers regarding FAM72A’s roles in B cell antibody diversification were published back-to-back in Nature [2,3]. With the utilization of a genome-wide CRISPR screen, Feng et al. found that FAM72A induced mutagenic DNA repair during antibody maturation by encountering UNG2. FAM72A promoted U·G mispairs, and B cells from Fam72a knockout mice exhibited somatic hypermutation [3]. Meanwhile, Rogier et al. also reported that FAM72A prompted diversification of the B cell receptor repertoire by facilitating error-prone DNA repair [2]. These findings suggest that FAM72A overexpression may accelerate mutagenesis in cancer. Previous bioinformatic analysis indicated that FAM72A was involved in lung adenocarcinoma and glioblastoma multiforme (GBM) [5,6]. However, the effects of enhanced FAM72A gene expression on prognosis have not been systematically evaluated across different cancer types.

Moreover, the tumor microenvironment (TME) is composed of various cellular components and an extracellular matrix, of which infiltrating immune cells account for a large proportion [7]. Tumor-immune cell interaction has been recognized to be a crucial determinator of tumor escape, progression, and therapeutic response. The pre-existing antitumor adaptive immune contexture involves, but is not limited to, antigen processing (e.g., HLAs), checkpoints/immunomodulators, effect cells (e.g., CD8+ T cells), and suppressor cells (e.g., Treg) [8]. Understanding the TME may help further explore the mechanism of tumor development and improve therapeutic efficacy. Given the crucial role of FAM72A in B cell antibody diversification, the relationship between FAM72A and the tumor immune environment deserves particular attention.

Overall, the studies on FAM72A are still in their infancy, and its exact functions in tumor development, progression, the tumor immune microenvironment, and response to anticancer treatment are unclear. Hence, we aim to conduct a pan-cancer analysis to understand the role of FAM72A across 33 cancer types. Several public databases, including The Cancer Genome Atlas (TCGA) and the Genotype-Tissue Expression (GTEx), were used to determine expression levels and prognostic implications of FAM72A from the perspective of pan-cancer. This pan-cancer analysis comprehensively evaluated the potential association of FAM72A with clinical outcomes, tumor mutation burden (TMB), MSI (microsatellite instability), immune score, intratumoral immune infiltrating cell subsets, and immune-related genes. Finally, we studied the biological function of FAM72A. The current study indicates that FAM72A has promise as a prognostic biomarker in various cancers, and FAM72A plays a vital role in the tumor immune microenvironment.

## 2. Results

### 2.1. Landscape of FAM72A mRNA Expression Levels in Pan-Cancer Tissues 

**Figure 1:**
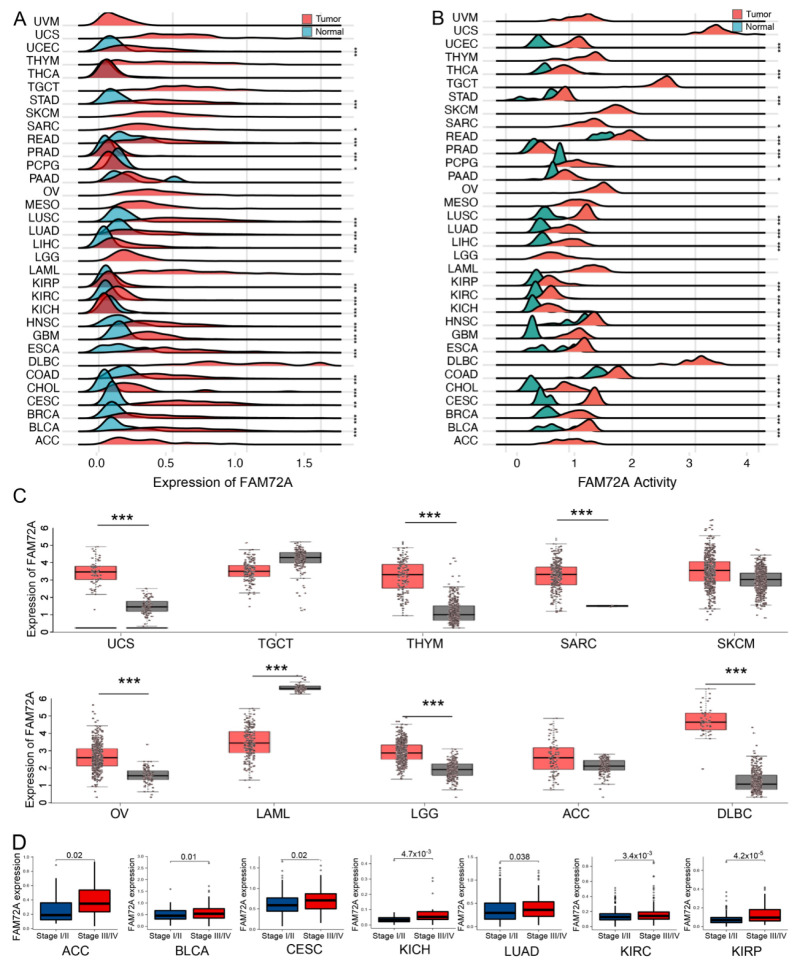
The expression of FAM72A in pan-cancer and different pathological stages. Differential expression (**A**) and activity (**B**) of FAM72A in normal and tumor samples of 33 tumors in The Cancer Genome Atlas (TCGA) database. (**C**). For tumors lacking corresponding normal tissue samples, the GEPIA web tool, incorporating normal tissues from the Genotype-Tissue Expression (GTEx) database, was used to compare FAM72A expression between the tumor and the normal tissues. (**D**). Expression of FAM72A in different pathological stages of indicated tumors. * *p* < 0.05, ** *p* < 0.01, and *** *p* < 0.001.

The flow chart of this research is shown in Supplementary Figure S1. Compared to normal tissues, significantly elevated mRNA expression levels of FAM72A were observed in 82% of 33 cancer types, including bladder urothelial carcinoma (BLCA), breast invasive carcinoma (BRCA), cervical squamous cell carcinoma and endocervical adenocarcinoma (CESC), cholangiocarcinoma (CHOL), colon adenocarcinoma (COAD), esophageal carcinoma (ESCA), GBM, lung squamous cell carcinoma (LUSC), lung adenocarcinoma (LUAD), and head and neck squamous cell carcinoma (HNSC) (Figure 1A,C). We next conducted single-sample gene set enrichment analysis (ssGSEA) with 100 FAM72A-correlated genes to calculate FAM72A activity. FAM72A activity was significantly higher in cancers than in normal tissue in tumors with increased FAM72A levels (Figure 1B). Since the activity score was derived from FAM72A-correlated genes, samples with high FAM72A expression levels are more likely to have high activity scores. We performed the correlation test for the expression and activity of FAM72A in several randomly selected tumor types, and positive correlations were observed (Supplementary Figure S2). Additionally, FAM72A expression levels increased with tumor progression in adrenocortical carcinoma (ACC), BLCA, CESC, kidney chromophobe (KICH), LUAD, kidney renal clear cell carcinoma (KIRC), and kidney renal papillary cell carcinoma (KIRP) (Figure 1D).

### 2.2. Correlation between FAM72A Expression Level and Cancer Prognosis 

**Figure 2:**
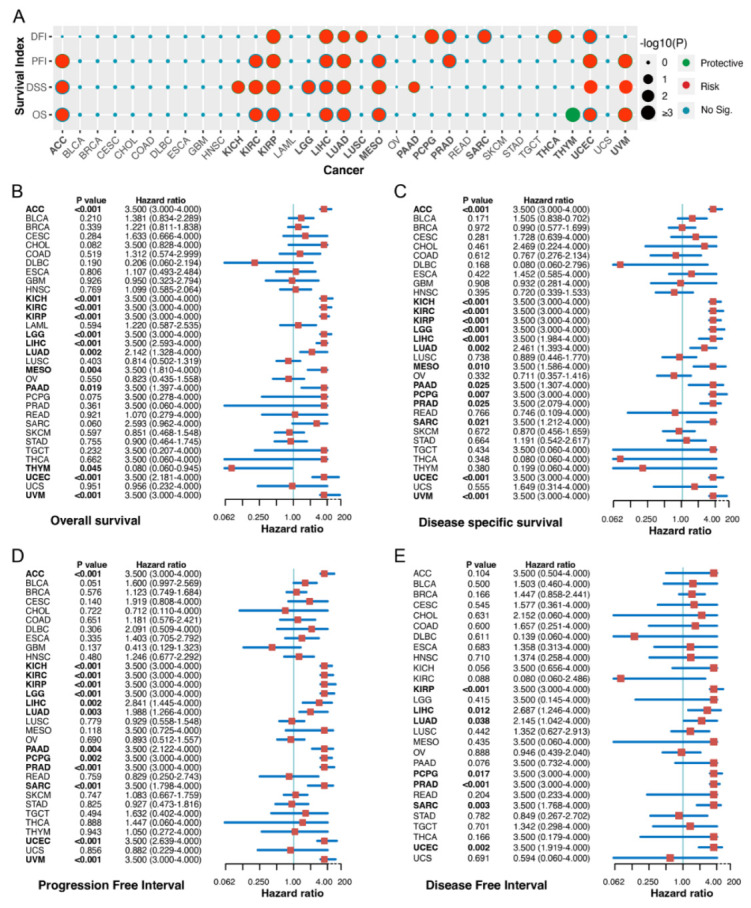
Correlation between the expression of FAM72A and prognosis in pan-cancer. (**A**). Kaplan- Meier survival analyses were performed to determine the association between FAM72A and several clinical endpoints, including overall survival (OS), disease-specific survival (DSS), disease-free interval (DFI), and progression-free interval (PFI) in 33 tumors. Statistical results were summarized and visualized using the “survival” and “ggplot2” R packages. The forest plots of univariate Cox regression analyses for FAM72A regarding OS (**B**), DSS (**C**), PFI (**D**), and DFI (**E**).

The clinical relevance of FAM72A in different tumor types was explored using Kaplan-Meier survival analysis. The results showed that high FAM72A levels were associated with poor OS in ACC, KIRC, KIRP, liver hepatocellular carcinoma (LIHC), LUAD, mesothelioma (MESO), uterine corpus endometrial carcinoma (UCEC), and uveal melanoma (UVM), but had a favorable OS with thymoma (THYM) (Figure 2A, Supplementary Figure S3). Moreover, a univariate Cox regression analyses identified high FAM72A as a risk factor for OS in ACC, KICH, KIRC, KIRP, brain lower grade glioma (LGG), LIHC, LUAD, MESO, pancreatic adenocarcinoma (PAAD), UCEC, and UVM, but a protective factor for overall survival (OS) in THYM (Figure 2B). Similar analyses were also performed against disease-specific survival (DSS), progression-free interval (PFI), and disease-free interval (DFI), (Figure 2C–E, Supplementary Figures S4–S6). These results suggest that FAM72A expression has a solid prognostic ability in different tumors.

### 2.3. Diagnostic Value of FAM72A for Pan-Cancer

**Figure 3:**
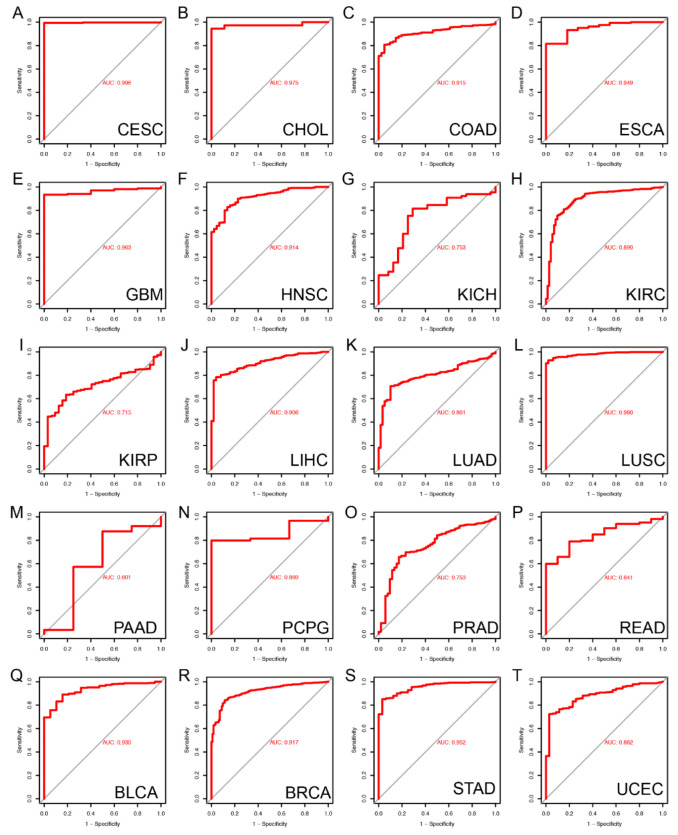
Accuracy of FAM72A in discriminating tumor from normal tissue in pan-cancer. (**A**–**T**). The area under the curve (AUC) of the receiver operating characteristic (ROC) for indicated tumors.

We also used receiver operating characteristic (ROC) to evaluate the discrimination ability of FAM72A expression levels between cancerous and normal tissues. The area under the curve (AUC) values for ROC analysis in each cancer are shown in Figure 3A–T. The AUC values suggested that FAM72A could reliably distinguish cancerous from normal tissues for various types of cancer, especially CESC (AUC = 0.996), CHOL (AUC = 0.975), COAD (AUC = 0.915), ESCA (AUC = 0.949), GBM (AUC = 0.963), HNSC (AUC = 0.914), KICH (AUC = 0.753), KIRC (AUC = 0.890), KIRP (AUC = 0.715), LIHC (AUC = 0.906), LUAD (AUC = 0.801), LUSC (AUC = 0.980), pheochromocytoma and paraganglioma (PCPG) (AUC = 0.860), prostate adenocarcinoma (PRAD) (AUC = 0.753), rectum adenocarcinoma (READ) (AUC = 0.841), BLCA (AUC = 0.930), BRCA (AUC = 0.917), stomach adenocarcinoma (STAD) (AUC = 0.952), UCEC (AUC = 0.882). 

### 2.4. Correlation between FAM72A and Tumor Microenvironment in Different Tumors 

**Figure 4:**
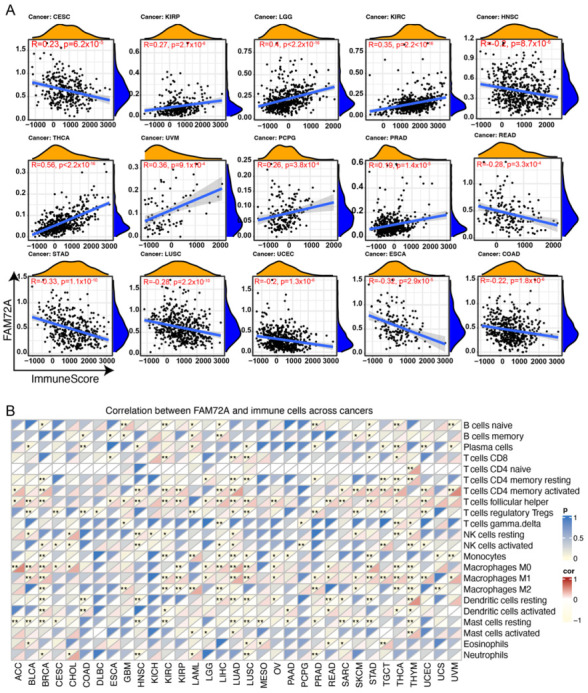
Correlation between the expression of FAM72A and immune cell infiltration in pan-cancer. (**A**). Correlation between the expression of FAM72A and ImmuneScore derived from the Estimation of Stromal and Immune cells in Malignant Tumor tissues using Expression data (ESTIMATE) algorithm. (**B**). Correlation between the expression of FAM72A and proportions of 22 infiltrating immune cell subsets evaluated using the Cell-type identification by Estimating Relative Subsets of RNA Transcripts (CIBERSORT) method. Correlations were determined by the Spearman method. * *p* < 0.05, and ** *p* < 0.01.

Intratumoral stromal and immune cells constitute the majority of tumor-associated normal cells. Not only immune cells but also stromal cells regulate tumor growth and progression. The Estimation of Stromal and Immune cells in Malignant Tumor tissues using Expression data (ESTIMATE) algorithm was used to evaluate infiltrating immune and stromal cells by calculating immune scores and stromal scores. FAM72A expression was found to positively correlate with the immune score in KIRC, KIRP, LGG, KIRC, thyroid carcinoma (THCA), UVM, and PCPG, but negatively correlated with the immune score in CESC, STAD, LUSC, UCEC, HNSC, READ, COAD, and ESCA (Figure 4A). Regarding stromal score, a positive correlation with FAM72A expression was seen in THCA, PCPG, PRA, acute myeloid leukemia (LAML), LGG, KIRP, KIRC, and a negative correlation in BRCA, UCEC, STAD, testicular germ cell tumors (TGCT), LUSC, and HNSC (Supplemental Figure S7A–M).

Previous studies suggest that FAM72A may be implicated in immunogenicity [2,3]. With this in mind, we explored the correlation between FAM72A expression and intratumoral immune cell infiltration. The Estimating Relative Subsets of RNA Transcripts (CIBERSORT) algorithm was used to determine the composition of 22 immune cell subsets based on gene expression profiles. 

Results indicated that FAM72A was significantly correlated with immune cell infiltration across different tumor types. For instance, FAM72A was significantly associated with ten or more immune cell subsets in BRCA, KIRC, LIHC, LUAD, LUSC, STAD, THCA, and THYM. FAM72A exhibited positive associations with CD8+ T cells in ESCA, KIRC, LUAD, LUSC, skin cutaneous melanoma (SKCM), and THCA, negative associations with M2 macrophages in BRCA, KIRC, KIRP, LIHC, SKCM, THCA, THYM, and uterine carcinosarcoma (UCS), as well as negative associations with Treg cells in BLCA, CESE, COAD, DLBC, LAML, LUAD, LUSC, PRAD, SKCM, TGCT, and UCES (Figure 4B). 

### 2.5. Correlations of FAM72A Expression with TMB, MSI, and Immune Checkpoint Genes in Cancers

**Figure 5:**
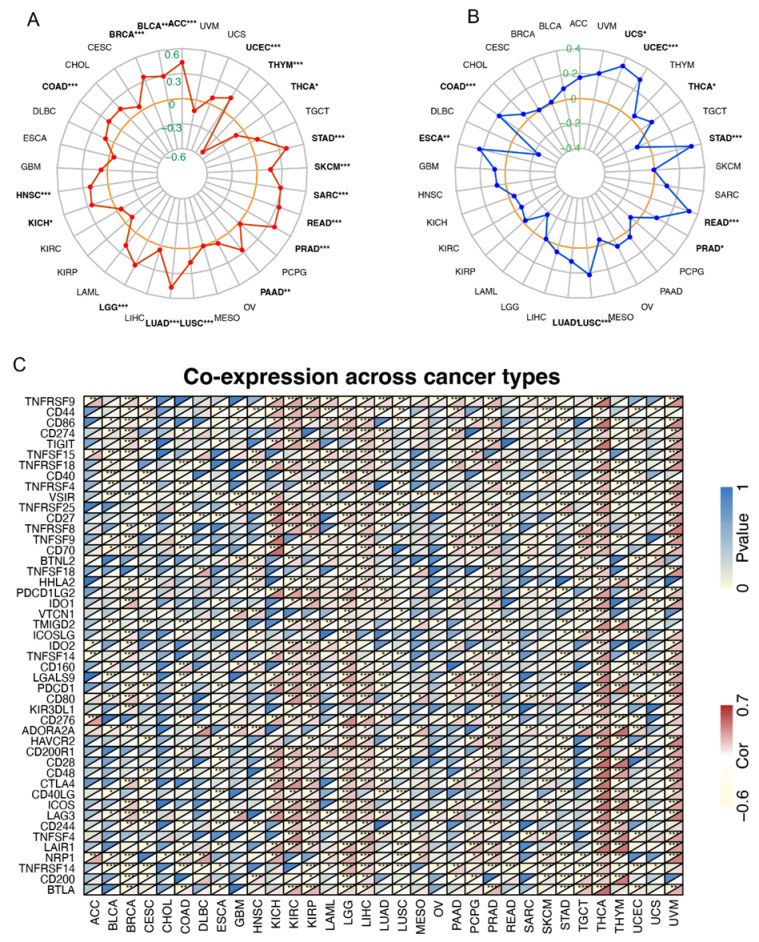
Correlation between the expression of FAM72A with tumor mutation burden (TMB), microsatellite instability (MSI), and immune checkpoint genes in pan-cancer. A-B. Radar maps showing the correlation of the expression of FAM72A with TMB (**A**) and MSI (**B**) by radargrams in pan-cancer. (**C**). Heatmap for the correlation of the expression of FAM72A with immune genes by heatmap in pan-cancer. The Spearman method was used to estimate the correlations. * *p* < 0.05, ** *p* < 0.01 and *** *p* < 0.001.

Both tumor mutation burden (TMB) and microsatellite instability (MSI) are pivotal characteristics of tumors and may be associated with response to immunotherapy. Therefore, it is indispensable to interrogate their association with FAM72A in pan-cancer. TMB is referred to as the count of somatic mutations per megabase of the queried genomic DNA fragments [9]. Our results are displayed in the radar map, with inner and outer gray circles representing the correlation coefficient of −0.6 to 0.6, respectively. Meanwhile, each red spot shows the correlation coefficient between FAM72A and TMB in cancers. FAM72A was significantly associated with the TMB in 18 cancer types, including ACC, BRCA, BLCA, COAD, HNSC, KICH, LGG, LUAD, LUSC, PAAD, PRAD, READ, sarcoma (SARC), SKCM, STAD, THCA, THYM, and UCEC (Figure 5A). Microsatellite instability (MSI) is defined as a pattern of hypermutation located in genomic microsatellites resulting from defective mismatch repair (dMMR) [10]. In the same way, our results demonstrated that FAM72A was positively correlated with MSI in COAD, ESCA, LUAD, LUSC, PRAD, READ, STAD, THCA, UCEC, and UCS, with gray circles depicting the correlation coefficient from −0.4 to 0.4 (Figure 5B). 

Immune checkpoint genes play essential roles in cancer immunotherapy, which are categorized into co-stimulators (e.g., CD80, CD28, ICOSLG), co-inhibitors (e.g., PDCD1LG2, CD274, VTCN1, CD276), ligands (e.g., TIGIT, PDCD1, TNFSF9, CD70, CD40LG), and receptors (e.g., TNFRSF9, TNFRSF4, TNFRSF14, LAG3, ICOS, BTLA, CD27) [11]. To better understand the immunological implication of FAM72A, we explore whether FAM72A expression is related to the expression pattern of immune checkpoints (ICPs) [12]. The correlation between FAM72A and immunomodulators is displayed in Figure 5C. Many ICP genes were positively correlated with FAM72A in pan-cancer, especially in KICH, KIRC, KIRP, LGG, LIHC, LUAD, PRAD, THCA, THYM, and UVM.

### 2.6. Relation between FAM72A and Immunophenoscore across Cancers

**Figure 6:**
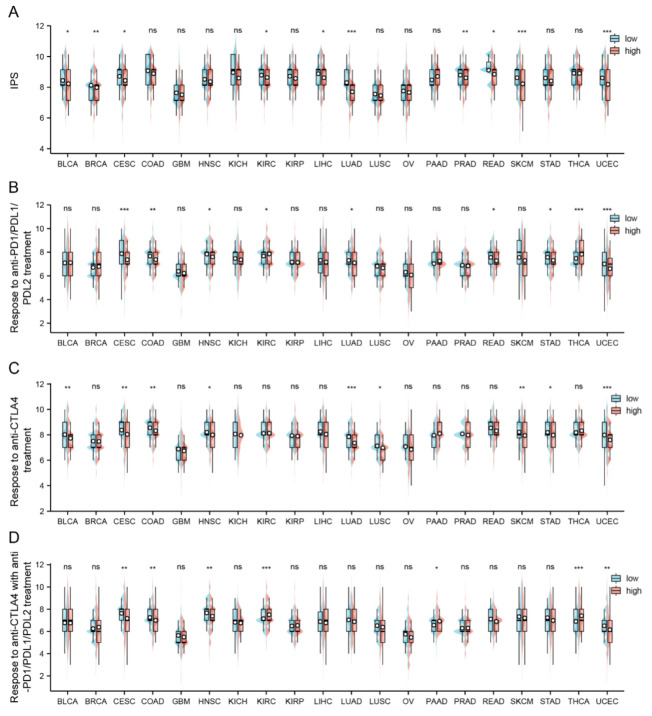
Assessment of immunotherapy response between FAM72A^high^ and FAM72A^low^ groups in pan-cancer. (**A**). Evaluation of immunophenoscore (IPS) for FAM72A^high^ and FAM72A^low^ groups, reflecting tumor immunogenicity. (**B**–**D**). the possibility of responding to anti-PD1/PDL1/PDL2 (**B**), anti-CTLA4 (**C**), and the combination (**D**) for the two groups across different cancers. ns, not significant, * *p* < 0.05, ** *p* < 0.01, and *** *p* < 0.001.

An immunophenoscore (IPS) was developed to comprehensively evaluate the tumor samples’ immunogenicity. Our results showed that the FAM72A^low^ group possessed a significantly higher IPS than the FAM72A^high^ group in BLCA, BRCA, CESC, KIRC, LIHC, LUAD, PRAD, READ, SKCM, and UCEC (Figure 6A). Moreover, the FAM72A^low^ group was more likely to gain benefits from anti-PD1/PDL1/PDL2 therapies in CESC, COAD, HNSC, LUAD, READ, STAD, UCEC (Figure 6B), and respond to anti-cytotoxic T-lymphocyte associated protein 4 (CTLA-4) therapy in BLCA, CESC, COAD, HNSC, LUAD, LUSC, SKCM, STAD, and UCEC (Figure 6C). Finally, the FAM72A^low^ group was prone to respond to the combination of the two immunotherapies in CESC, COAD, HNSC, and UCEC (Figure 6D). On the other hand, the FAM72A^high^ group has a tendency to respond to anti-PD1/PDL1/PDL2 therapies in KIRC and THCA (Figure 6B), and respond to the combined immunotherapies in KIRC, PAAD, and THCA (Figure 6D). These findings further validated the immunological implication of FAM72A in cancer.

### 2.7. The Signaling Pathway Associated with FAM72A

**Figure 7:**
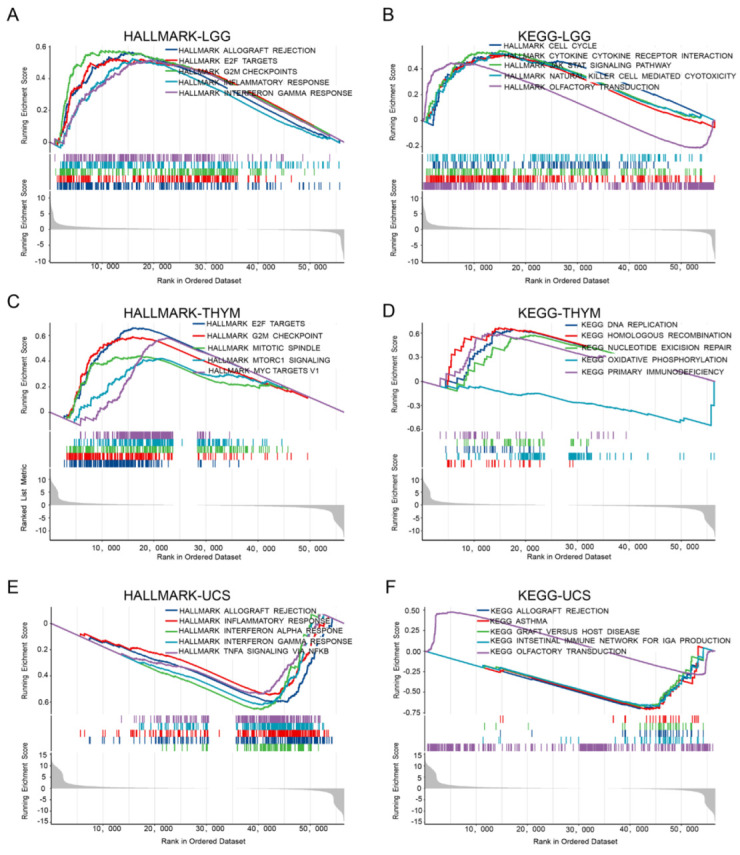
Gene set enrichment analysis (GSEA) regarding FAM72A for cancers. (**A**–**F**). Enrichment results of HALLMARK in LGG (**A**), THYM (**C**), and UCS (**E**). The enriched KEGG signaling pathways in LGG (**B**), THYM (**D**), and UCS (**F**).

To investigate the potential function of FAM72A Pan-cancer, we carried out a gene set enrichment analysis (GSEA). The top five signaling pathways significantly associated with FAM72A are exhibited in Figure 7. In LGG, FAM72A was associated with inflammatory response, interferon-gamma response, cell cycle, cytokine-cytokine receptor interaction, JAK-STAT signaling, and nature killer cell-mediated cytotoxicity (Figure 7A,B). In THYM, FAM72A was associated with G2M checkpoint, MTORC1 signaling, MYC targets v1, DNA replication, two DNA repair pathways, oxidative phosphorylation, and primary immunodeficiency (Figure 7C,D). In UCS, FAM72A was associated with inflammatory response, interferon α response, interferon γ response, and TNFα signaling via NF-κB (Figure 7E,F). These results suggest that FAM72A plays a fundamental role in tumorigenesis. 

### 2.8. In Vitro Validation

**Figure 8:**
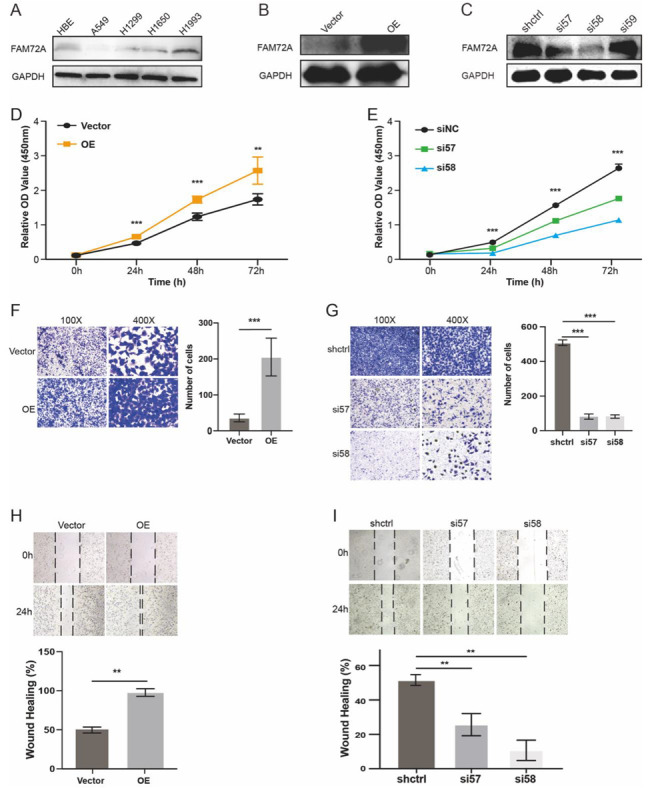
Validation of the potential function of FAM72A in tumors by in vitro assays. (**A**). Differential expression of FAM72A in normal lung bronchial epithelial cells and lung cancer cells by Western blot analysis. (**B**,**C**). Examination of FAM72A expression in A549 (**B**) and H1993 cells (**C**) stably infected lentiviral vectors overexpressing FAM72A and lentiviral vectors carrying shFAM72A, respectively. The overexpression of FAM72A promotes the proliferation of A549 cells by CCK-8 assay (**D**), Transwell assay (**F**), and wound healing test (**H**). Knockdown of FAM72A inhibits the proliferation of H1993 cells by CCK-8 assay (**E**), Transwell assay (**G**), and wound healing test (**I**). ** *p* < 0.01, and *** *p* < 0.001.

We first examine the endogenous expression levels of FAM72A in normal lung and cancer cell lines. A549 cells with relatively low levels of FAM72A were used for gain-of-function analysis, while H1993 cells were adopted for loss-of-function analysis (Figure 8A–C). The enforced expression of FAM72A promoted lung cancer cell proliferation as indicated by a CCK assay (Figure 8D). Moreover, Transwell migration and wound healing assays demonstrated enhanced migration in cells overexpressing FAM72A (Figure 8F,H). On the contrary, FAM72A depletion demonstrated the opposite effects on the proliferation (Figure 8E) and migration of H1993 cells (Figure 8G,I). These results verified FAM72A’s oncogenic potential in lung cancer. 

## 3. Discussion 

### 3.1. Expression and Prognostic Implication

A pan-cancer analysis revealed that compared to normal tissues, significantly elevated mRNA expression levels of FAM72A were observed in 82% of cancers, including BLCA, BRCA, CESC, CHOL, COAD, ESCA, GBM, HNSC, KICH, KIRC, KIRP, LIHC, LUSC, LUAD, PCPG, PRAD, READ, SARC, STAD, and UCES. Furthermore, FAM72A expression levels increased with tumor progression in ACC, BLCA, CESC, KICH, LUAD, KIRC, and KIRP. In terms of prognosis, Kaplan-Meier survival and univariate Cox regression analysis demonstrated that patients with FAM72A^high^ tumors were more likely to suffer inferior survival in ACC, KICH, KIRC, KIRP, LGG, LIHC, LUAD, MESO, PAAD, UCEC, and UVM. 

To date, the evidence of FAM72A’s implications in cancer is very preliminary and lacks experimental validation. Guo et al. initially reported the elevated expression of FAM72A in some common cancer types, including colon (n = 34 pairs of tumor and matched normal tissues), breast (n = 50), lung (n = 21), uterus (n = 42), and ovary (n = 14) [1]. Furthermore, several TCGA-based bioinformatic studies indicated that FAM72A might be involved in cancer. FAM72 consists of four human-specific paralogs (A–D). For instance, the expression of the Ki67 gene (MKI67), the putative proliferation marker, was found to be tightly associated with levels of FAM72A, B, and D across different cancers [5]. Yu and colleagues explored the prognostic value of FAM72A–D and found that all the members were overexpressed in LUAD and showed potential as prognostic markers [6]. Recently, a prognostic classifier of 10 mitochondrial-related genes was identified in hepatocellular carcinoma (HCC), including ACOT7, ADPRHL2, ATAD3A, BSG, FAM72A, PDK3, PDSS1, RAD51C, TOMM34, and TRMU) [13]. Interestingly, Gao et al. recently developed an angiogenesis factors-based prognostic signature in hepatocellular carcinoma, in which FAM72 is also one of six key genes (GRM8, SPC25, FSD1L, SLC386A, FAM72A, and SLC39A10) [14]. Zhou and colleagues validated the upregulation of FAM72A in tumor samples of both HCC patients and a mouse HCC model [15]. These results suggest that FAM72A is a promising prognostic marker in multiple cancer types and deserves extensive investigation. 

### 3.2. Tumor Microenvironment

The tumor microenvironment (TME) is composed of tumor cells, stromal cells (e.g., fibroblasts and endothelial cells), a variety of immune cells (including T lymphocytes, NK cells, macrophages, and dendritic cells), and extracellular matrix (e.g., biochemical components released by these cells) [16]. These cells crosstalk with each other and collectively determine tumor fates, regression, or progression. TME is not only crucial for tumor proliferation, invasion, and metastasis but also affects the therapeutic effect [17]. With the development of high-throughput sequencing technology and machining learning, ESTIMATE was generated to infer stromal and immune cells in tumors based on RNA-sequencing data [18]. We adopted this method to determine stromal and immune scores for samples across 33 cancer types and found that immune scores derived from the ESTIMATE algorithm were differentially correlated to FAM72A among different cancer types, and this was also the case for stromal cells.

We also used the CIBERSORT algorithm to estimate the proportion of 22 immune cell subsets in each tumor sample. The correlation test indicated that FAM72A was extensively correlated to immune cell infiltrates in BRCA, KIRC, LIHC, LUAD, LUSC, STAD, THCA, and THYM. However, the association of FAM72A with cytotoxic effector cells (e.g., CD8+ T and NK cells) or repressive immune cells (e.g., M2 macrophages and Treg cells) varied among different tumors. These results indicated that the role of FAM72 in tumor immunogenicity is tissue specific.

### 3.3. Correlation to TMB, MSI, and ICPs

Given the tight correlation between FAM72A and TME, we further explored whether FAM72 is related to the response to immunotherapy. A growing body of evidence suggests that MSI, TMB, and programmed cell death protein 1 (PD-1)/programmed death-ligand 1 (PD-L1) expression levels are associated with an elevated response rate to immunotherapy and can facilitate the identification of patients suitable for immunotherapy. Our pan-cancer analysis revealed that FAM72A was significantly associated with the TMB in 18 cancer types, including ACC, BLCA, BRCA, COAD, HNSC, KICH, LGG, LUAD, LUSC, PAAD, PRAD, READ, SARC, SKCM, STAD, THCA, THYM, and UCEC. High TMB is consistently used to select proper patients for ICI therapy because somatic mutations in tumor DNA may have a chance to produce neoantigen-containing peptides, which can be processed, displayed onto major histocompatibility complex (MHC) molecules, and recognized by T cells. Although mutation-derived neoantigens are very rare, theoretically, the more somatic mutations a tumor harbors, the more immunogenic neoantigens that can be generated [19]. TMB variations have been characterized across different malignancies. TMB can represent a valuable estimation of tumor neoantigen load. To date, TMB is believed to be a key source of immunogenic neuropeptides presented on the MHC of the tumor cell surface, affecting patient response to ICIs [9]. Response to ICIs in solid tumors with high TMB have been observed in various tumors, including NSCLC, melanoma, and bladder cancer [20]. Mechanistic studies suggested that the reason that TMB predicts ICIs sensitivity might be attributed to TMB-mediated neoantigens and tumor immunogenicity [20].

Our results indicated positive correlations between FAM72A and MSI in COAD, ESCA, LUAD, LUSC, PRAD, READ, STAD, THCA, UCEC, and UCS. Mutations in MLH1, MSH2, MSH6, and PMS2 genes often lead to dMMR. Therefore, MSI is also considered a marker of dMMR. MSI has been observed among 27 tumor types [21]. For instance, MSI has been considered a primary predictive marker for the responses to ICIs in CRC, including nivolumab or pembrolizumab targeting PD-1 [22]. 

Moreover, many ICP genes were positively correlated with FAM72A in pan-cancer, especially in KICH, KIRC, KIRP, LGG, LIHC, LUAD, PRAD, THCA, THYM, and UVM. Notably, CD274/PD-L1, PDCD1LG2/PD-L2, PDCD1/PD1, cytotoxic T lymphocyte-associated antigen 4 (CTLA4), T cell membrane protein 3 (TIM3; also known as HAVCR2), and lymphocyte activation gene 3 (LAG3) were correlated to FAM72A across different cancers.

ICIs targeting PD-1/PD-L1 and CTLA-4 have caused a revolution in cancer care by reversing the immunosuppressive tumor microenvironment. Inhibitors of other ICP genes are also underway, such as IDO1 and TIGIT, which also showed correlations with FAM72A in multiple tumors [23,24,25]. Indoleamine 2,3-dioxygenase (IDO) 1, an enzyme converting tryptophan to kynurenine, is an immunosuppressant in TME. Blockade of IDO1 is a promising strategy to reignite antitumor immunity [24]. T Cell Immunoreceptor With Ig And ITIM Domains (TIGIT) belongings to the V-Set and immunoglobulin domain containing (VSIG) family. As a coinhibitory receptor during T-cell activation, TIGIT has received considerable attention as a putative immune checkpoint in TME [25]. The double blocking of the TIGIT and PD-1/PD-L1 pathway showed synergistic antitumor effects in colorectal cancer and ovary cancer by upregulating the effector activity of T cells and NK cells [23]. 

Furthermore, we found that FAM72A^low^ tumors exhibited a significantly higher IPS than the FAM72A^high^ tumors. Moreover, the FAM72A^low^ group was more likely to benefit from anti-PD1/PDL1/PDL2, anti-CTLA4, and combined immunotherapies in various cancers. The IPS is the most comprehensive estimator of tumor immunogenicity. It is calculated by incorporating four key tumor immunogenicity determining factors, including effector cells, checkpoints/immunomodulators, antigen processing major histocompatibility complex (MHC) molecules (e.g., HLAs), and immunosuppressive cells. The immunophenoscore has shown remarkable performance in terms of predicting response to immunotherapies blocking CTLA and PD1 [8]. Overall, these results indicated that FAM72A might be related to the response to ICP therapies. 

### 3.4. Functional Analysis

Finally, we performed in vitro experiments to verify the function of FAM72A in lung cancer. The overexpression of FAM72A promoted proliferation and colony formation and also facilitated the EMT and migration of lung cancer cells. The functional analysis of FAM72A is currently very limited. Guo et al. first identified FAM72A and demonstrated its overexpression in several commonly diagnosed malignancies [1]. They also found that FAM72A interacted with UNG2, a crucial component in the base excision repair pathway, and thereby proposed that FAM72A might participate in tumorigenesis via the BER pathway [1]. Wang et al. reported that enforced expression of FAM72A relieved H_2_O_2_-induced reactive oxygen species production and mitochondria membrane potential (Δψ) loss in nasopharyngeal carcinoma (NPC) cell lines. FAM72A also stimulated cell cycle progression in the NPC cells [26]. On the contrary, FAM72A depletion in the NPC tumor cell lines significantly reduced the cell population at the G1/S phase but increased the number of cells in the multiploid phase [26]. Recently, FAM72A was shown to induce error-prone DNA repair and assist in mutagenic repair in the process of antibody maturation [2,3]. These results suggested that FAM72A may be a new therapeutic target in cancer. Moreover, Zhou et al. observed that FAM72A depletion suppressed proliferation and inactivated the mTOR signaling in HCC cell lines [15]. 

Our results are largely dependent on analyzing public databases. Due to the striking advances in high-throughput technology, tons of big data have been generated, coupled with the exciting development of machine learning algorithms. Online databases are mainly based on big data, specific algorithms, and programming languages, which are easy to operate and have a high degree of visualization, rich functions, and fast updating speed. Big data lead to a revolution in various scientific communities, such as life science. For instance, The Cancer Genome Atlas (TCGA) unveiled the genomic landscape of human malignancy. The findings derived from mining data of TCGA projects have dramatically accelerated the understanding of tumor biology, the discovery of predictive biomarkers for early diagnosis and prognosis, as well as the identification of therapeutic targets. 

It should be noted that results derived from public data and statistical algorithms may unavoidablely suffer from the significant level of heterogeneity and the possible effect of bias. Findings from a bioinformatics analysis should be interpreted cautiously and cannot be translated directly into the clinical setting. However, they are informative and suggestive for researchers in the field. Our findings can be used as a source for other researchers interested in the role of FAM72A in cancers and to attract more attention to this potentially functionally important molecule. After all, we also performed a functional analysis to validate the oncogenic role of FAM72A on lung adenocarcinoma. 

In conclusion, our findings indicated that FAM72A might be a potential biomarker predicting prognosis and tumor immunogenicity in different tumors. High FAM72 might predict poor prognosis in ACC, KICH, KIRC, KIRP, LGG, LIHC, LUAD, MESO, PAAD, UCEC, and UVM, but a favorable prognosis in THYM. Moreover, high FAM72 might predict low immunogenicity in BLCA, BRCA, CESC, KIRC, LIHC, LUAD, PRAD, READ, SKCM, and UCEC. Functional analysis demonstrated that FAM72A played a tumor-promoting role in lung adenocarcinoma.

## 4. Materials and Methods 

### 4.1. Data Collection

RNA sequencing data for 33 types of cancers were obtained from The Cancer Genome Atlas (TCGA) Data Portal (https://portal.gdc.cancer.gov/, accessed on 15 October 2022) [27]. Accordingly, we acquired clinical data for patients from the UCSC Xena website (https://xena.ucsc.edu/, accessed on 17 October 2022), including OS, DSS, DFI, and PFI [28]. First, the expression levels of FAM72A in normal and tumor tissues were compared using the Wilcoxon rank sum test function in the “ggplot2” R package. Moreover, an online tool, the Gene Expression Profiling Interactive Analysis (GEPIA) (http://gepia.cancer-pku.cn/, accessed on 19 October 2022), was adopted to compare FAM72A expression between tumorous and normal tissues for several tumors lacking enough normal tissues [29]. GEPIA comprised gene expression information from TCGA and gene expression profiles for normal tissues from the Genotype-Tissue Expression (GTEx) database (https://www.genome.gov/Funded-Programs-Projects/Genotype-Tissue-Expression-Project, accessed on 22 October 2022). FAM72A expression was determined for stages I + II and stage III + IV tumors to explore whether its expression increased with tumor progression [29].

### 4.2. Estimation of FAM72A Activity in Patient Samples

We used a metagene approach to assess FAM72A activity in patient samples, as previously published [30,31,32]. A Spearman correlation test was performed to select the top 100 genes mostly correlated with FAM72A through the TCGA datasets, which were used to represent the activity of FAM72A. The ssGSEA was utilized to evaluate FAM72A activity in each sample. The analyses were executed using the “gene set variation analysis (GSVA)” and “GSEABase” R packages based on 100 genes.

### 4.3. Prognostic Analysis

The clinical endpoints selected for this study included OS, DSS, PFI, and DFI. OS was referred to as the time from diagnosis to death, regardless of causes. Unlike OS, DSS does not count patients who died from causes aside from a specific disease. PFI is a period free of disease progression or death from any cause. Patients who died from causes except for a specific disease are excluded from DFI. Patients were divided into FAM72A^low^ and FAM72A^high^ groups using the minimum *p*-value method. A Kaplan-Meier survival analysis was performed to compare the clinical outcomes of patients with FAM72A^low^ or FAM72A^high^ tumors with the application of “survival” and “survminer” R packages. We also implemented univariate Cox regression analysis to estimate the prognostic value of FAM72A by calculating the hazard ratio (HR) and 95% confidence interval (CI) by executing the “survival” and “foresplot” R packages [33].

### 4.4. FAM72A’s Capacity to Distinguish Tumor from Non-Tumor Tissues

A ROC analysis of FAM72A expression levels was performed using the “pROC” R package to examine whether FAM72A expression levels can separate tumors and normal tissue across the 33 types of cancer, and the area under the curve (AUC) was calculated [34]. An AUC value greater than 0.7 is considered acceptable. 

### 4.5. Implication of FAM72A Expression in Tumor Immune Microenvironment

The ESTIMATE algorithm [18] was employed to determine the immune score and stromal score with the application of the “ESTIMATE” package in R software. The correlation between FAM72A expression and immune score or stromal score was interrogated in 33 tumors using the Spearman method (*p* < 0.05 and R > 0.1). 

We further dissected the tumor-infiltrating immune cells that constitute a crucial component of tumor tissues. Accumulating evidence demonstrated their clinicopathologic significance in predicting prognosis and therapeutic efficiency [35]. Therefore, we explored the relevance between FAM72A and immune infiltrates in this study. The CIBERSORT algorithm was applied to assess the levels of 22 infiltrating immune cell subtypes. These cells included naive B cells, memory B cells, plasma cells, CD8 T cells, naive CD4 T cells, resting memory CD4 T cells, memory-activated CD4 T cells, follicular helper T cells, T regulatory cells (Tregs), gamma delta T cells, resting NK cells, activated NK cells, monocytes, M0 macrophages, M1 macrophages, M2 macrophages, resting dendritic cells, activated dendritic cells, resting mast cells, activated mast cells, and eosinophils [36,37]. The R packages “limma” and “CIBERSORT” were used. The Spearman method was performed to determine the correlation of FAM72A with 22 immune cell subsets in pan-cancer, and the R package “ggplot2” was used to generate the correlation matrix heatmap.

### 4.6. Correlation Analysis of FAM72A with TMB, MSI, Checkpoint Genes, and Immunophenoscore

TMB measures the mutation number in a specific cancer genome. Numerous studies have explored the significance of using TMB as a biomarker for identifying patients sensitive to checkpoint inhibitors [38]. We downloaded the somatic mutation data for all TCGA patients (https://tcga.xenahubs.net, accessed on 23 October 2022) and calculated TMB scores for each sample using the “maftools” R package. Microsatellite instability (MSI) is featured by the widespread length polymorphisms of microsatellite sequences resulting from DNA polymerase slippage [39]. MSI is used as an indicator of genetic instability for the cancer detection index, and patients with high-MSI cancers have been shown to benefit from immunotherapy [40]. The MSI scores of 33 tumors were obtained from the published literature [41]. Spearman’s rank method was used to determine the correlation of FAM72A with TMB and MSI. The correlation results for TMB and MSI were visualized in radar maps.

Due to the putative roles of immune checkpoints ICPs in cancer immunity, we interrogated the association of 47 ICP genes with FAM72A, including co-stimulators (CD80, CD28, ICOSLG), co-inhibitors (PDCD1LG2, CD274, VTCN1, CD276), ligands (TIGIT, PDCD1, TNFSF9, CD70, CD40LG), receptors (TNFRSF9, TNFRSF4, TNFRSF14, LAG3, ICOS, BTLA, CD27), CD44, CD86, TNFSF15, VSIR, TNFRSF25, TNFRSF8, BTNL2, TNFSF18, HHLA2, TMIGD2, IDO2, TNFSF14, CD160, LGALS9, KIR3DL1, ADORA2A, HAVCR2, CD200R1, CD48, CTLA4, CD244, TNFSF4, LAIR1, NRP1, CD200, and IDO1 [12].

Charoentong and colleagues calculated the immunophenoscore (IPS) for 20 types of cancer using the TCGA database and deposited them in The Cancer Immunome Atlas (TCIA, https://tcia.at/home, accessed on 24 October 2022) [8]. The IPS varies from 0 to 10, with 0 and 10 indicating the lowest and highest degree of immunogenicity. We made comparisons of IPS for the FAM72A^low^ and FAM72A^high^ groups and subsequently evaluated their responses to anti-PD1/PDL1/PDL2 and anti-CLA4 treatments.

### 4.7. Gene Set Enrichment Analysis in Pan-Cancer

To explore the biological signaling pathways, the gene sets h.all.v7.4.symbols.gmt and c2.cp.kegg.v7.4.symbols.gmt were downloaded from the GSEA website (https://www.gsea-msigdb.org/gsea/downloads.jsp, accessed on 27 October 2022) [42]. FAM72A median values were used to divide patients into FAM72A^high^ and FAM72A^low^ groups. A GSEA was performed for pan-cancer. The enrichment results were analyzed and plotted using the R package “limma”, “org.Hs.eg.db”, “clusterProfiler”and “enrichplot”. Results with |NES| > 1, *p* < 0.05, and FDR q < 0.25 were considered significant. The top five significantly enriched signaling pathways were demonstrated.

### 4.8. Cell Culture

The Human lung bronchial epithelial (Beas-2B) and four human NSCLC cell lines (A549, H1299, H1650, and H1993) were purchased from the Cell Bank of the Chinese Academy of Science (Shanghai, China). Cells were cultured in the DMEM (Beas-2B), or RPMI-1640 (A549, H1299, H1650, and H1993) medium (Invitrogen Corporation, Carlsbad CA, USA) supplemented with 10% fetal bovine serum (PAN-Biotech, Aidenbach Germany), penicillin G (100 U/mL, Beyotime, China) and streptomycin (100 μg/mL, Corning Inc. Corning, NY, USA). Cell cultures were kept in a humidified incubator at 37 °C with 5% CO_2_.

### 4.9. Establishment of Stable Cell Lines

We first purchased lentiviral vectors for cDNA of the entire human FAM72A coding sequence, and empty vectors (Ubi-MCS-3FLAG-CBh-gcGFP-IRES-puromycin) from Genechem Co., Ltd. (Shanghai, China). Meanwhile, lentiviral vectors carrying shFAM72A (LV-FAM72A-RNAi (114657-1), LV-FAM72A-RNAi (114658-2), LV-FAM72A-RNAi (114659-2)), and shcontrol (shctrl) (hU6-MCS-CBh-gcGFP-IRES-puromycin) were also obtained from the same company. The sequences of the three shRNA targeting FAM72A were as follows: 5′-CCAGGCAGTTTATGATATTAA-3′, 5′-GCAGTGGACTTCACTGGAAGA-3′, and 5′-GCCAGAGATAGAAGAGAGTAC-3′ (Genechem Co., Ltd., Shanghai, China). A549 and H1993 cells were parallelly cultured in 6-well plates. While reaching 60~70% confluence, 10 ul of the lentiviruses overexpressing FAM72A were added to each well for A549 cells and incubated for 36 h; likewise, a similar number of each type of shFAM72A viruses were used to infect the H1993 cell. Cells were then maintained in a culture medium with the addition of puromycin (Labgic Technology Co., Ltd., Beijing, China) to establish stable cell lines. Finally, a Western blot was used to examine FAM72A expression in cells.

### 4.10. Western Blotting

Cells were harvested, washed twice with phosphate-buffered saline (PBS, Procell, Wuhan, China), and lysed at 4 °C with RIPA (Solarbio, Beijing, China) buffer supplemented with a proteinase inhibitor cocktail (Solarbio, Beijing, China) for 20 min. Equal amounts of proteins were electrophoresed in SDS-PAGE (15%) and transferred to PVDF membranes. After blocking with 5% skimmed milk in PBST at room temperature for 1 h, the membranes were incubated overnight at 4 °C with primary antibodies against FAM72A (1:1000, Proteintech) and GAPDH (1:1000, Absin), as needed. After washing with PBST, the membranes were incubated with a secondary antibody (1:1000, Beijing Zhongshan Golden Bridge Biotechnology Co. Ltd., Beijing, China) for 1 h at room temperature. Finally, an ECL detection system (Tanon, Shanghai) was used to detect targeted protein bands. GAPDH was used as an internal control.

### 4.11. Cell Proliferation

We seeded 3 × 10^3^ cells into each well of 96-well plates with the addition of 100 μL of culture medium. Cells grew in an incubator under standard conditions, and a CCK-8 assay (Abbkine Scientific Co, Wuhan, China) was performed at 24 h, 48 h, and 72 h after initial attachment. The OD values were measured at 450 nm with a microplate reader.

### 4.12. Cell Migration Assay

Cell migration was assessed using 8-μm-pore Transwell compartments (Corning Inc, Corning NY, USA). Cell suspensions (3 × 10^4^ cells) in serum-free medium were added to the upper compartment. After cells were incubated at 37 °C for 24 h, the translocated cells were fixed by 4% paraformaldehyde for 20 min and then stained with 0.5% crystal violet for 20 min at room temperature. Cells were counted under a light microscope (Nikon, Tokyo, Japan).

### 4.13. Wound-Healing Assay

We also evaluated the effects of FAM72A on cell migration using the wound-healing assay. Briefly, a wound was generated in a 6-well plate by scratching the surface with a 200 μL pipette tip. The wounded areas were photographed under a light microscope (Nikon, Tokyo, Japan) when the wound was created (0 h) and 24 h later. The percentage of wound healing was calculated using the following formula: [1 − (empty area 24 h/empty area 0 h)] × 100%.

### 4.14. Statistical Analysis

All bioinformatic analyses were carried out with the R software version 4.1.3 (www.r-project.org, accessed on 15 October 2022). The ESTIMATE and CIBERSORT algorithms were adopted to assess tumor immune environments. Immunophenoscore (IPS) was utilized to estimate tumor immunogenicity and response to immune checkpoint inhibitors (ICIs). The Spearman method was conducted to determine the correlation of FAM72A with the immune score, infiltrating immune cells, tumor mutation burden (TMB), microsatellite instability (MSI), checkpoint genes, and IPS. R packages (limma, ggplot2, CIBERSORT, ESTIMATE, GSVA, CIBERSORT, org.Hs.eg.db, clusterProfiler, DOSE, and enrichplot) were implemented to visualize the results. Statistics for the CCK8, wound healing, and Transwell migration assays were analyzed using Graphpad Prism 9.0 software (GraphPad, La Jolla, CA, USA). Differences in proliferation and migration between vector and experimental groups were checked using the Student’s t-test. The significance threshold was set as 0.05 for all statistical analyses.

## Data Availability

Not applicable.

## Supplementary Materials

The following supporting information can be downloaded at: https://www.mdpi.com/article/10.3390/ijms24010375/s1.

## Abbreviations

FAM72A: Family with sequence similarity 72 Member A; UNG2, uracil DNA-glycosylase 2; dU, deoxyuracil; dC, deoxycytidine; ACC, adrenocortical carcinoma; BLCA, bladder urothelial carcinoma; BRCA, breast invasive carcinoma; CESC, cervical squamous cell carcinoma and endocervical adenocarcinoma; CHOL, cholangiocarcinoma; COAD, colon adenocarcinoma; DLBC, lymphoid neoplasm diffuse large b-cell lymphoma; ESCA, esophageal carcinoma; GBM, glioblastoma multiforme; HNSC, head and neck squamous cell carcinoma; KICH, kidney chromophobe; KIRC, kidney renal clear cell carcinoma; KIRP, kidney renal papillary cell carcinoma; LAML, acute myeloid leukemia; LGG, brain lower grade glioma; LIHC, liver hepatocellular carcinoma; LUAD, lung adenocarcinoma; LUSC, lung squamous cell carcinoma; MESO, mesothelioma; OV, ovarian serous cystadenocarcinoma; PAAD, pancreatic adenocarcinoma; PCPG, pheochromocytoma and paraganglioma; PRAD, prostate adenocarcinoma; READ, rectum adenocarcinoma; SARC, sarcoma; SKCM, skin cutaneous melanoma; STAD, stomach adenocarcinoma; TGCT, testicular germ cell tumors; THYM, thymoma; THCA, thyroid carcinoma; UCS, uterine carcinosarcoma; UCEC, uterine corpus endometrial carcinoma; UVM, uveal melanoma; OS, overall survival; DSS, disease-specific survival; DFI, disease-free interval, PFI, progression free interval; IPS, Immunophenoscore; TMB, tumor mutation burden; TME, the tumor microenvironment; MSI, microsatellite instability; ICPS, immune checkpoints; ROC, receiver operating characteristic; ICIs, immune checkpoint inhibitors; TCGA, The Cancer Genome Atlas; GTEx, Genotype-Tissue Expression; GEPIA, Gene Expression Profiling Interactive Analysis; GSEA, gene set enrichment analysis; ssGSEA, single sample gene set enrichment analysis, GSVA, Gene set variation analysis.
