# Supplementary material for: Prognostic and Immunological Implications of FAM72A in Pan-Cancer and Functional Validations
Source: Int J Mol Sci. 2022 Dec 26;24(1):375. doi: 10.3390/ijms24010375 (PMC9820597; doi:10.3390/ijms24010375)
Supplement: Supplementary file 1 [file ijms-24-00375-s001.zip › ijms-2073493-supplementary.pdf]

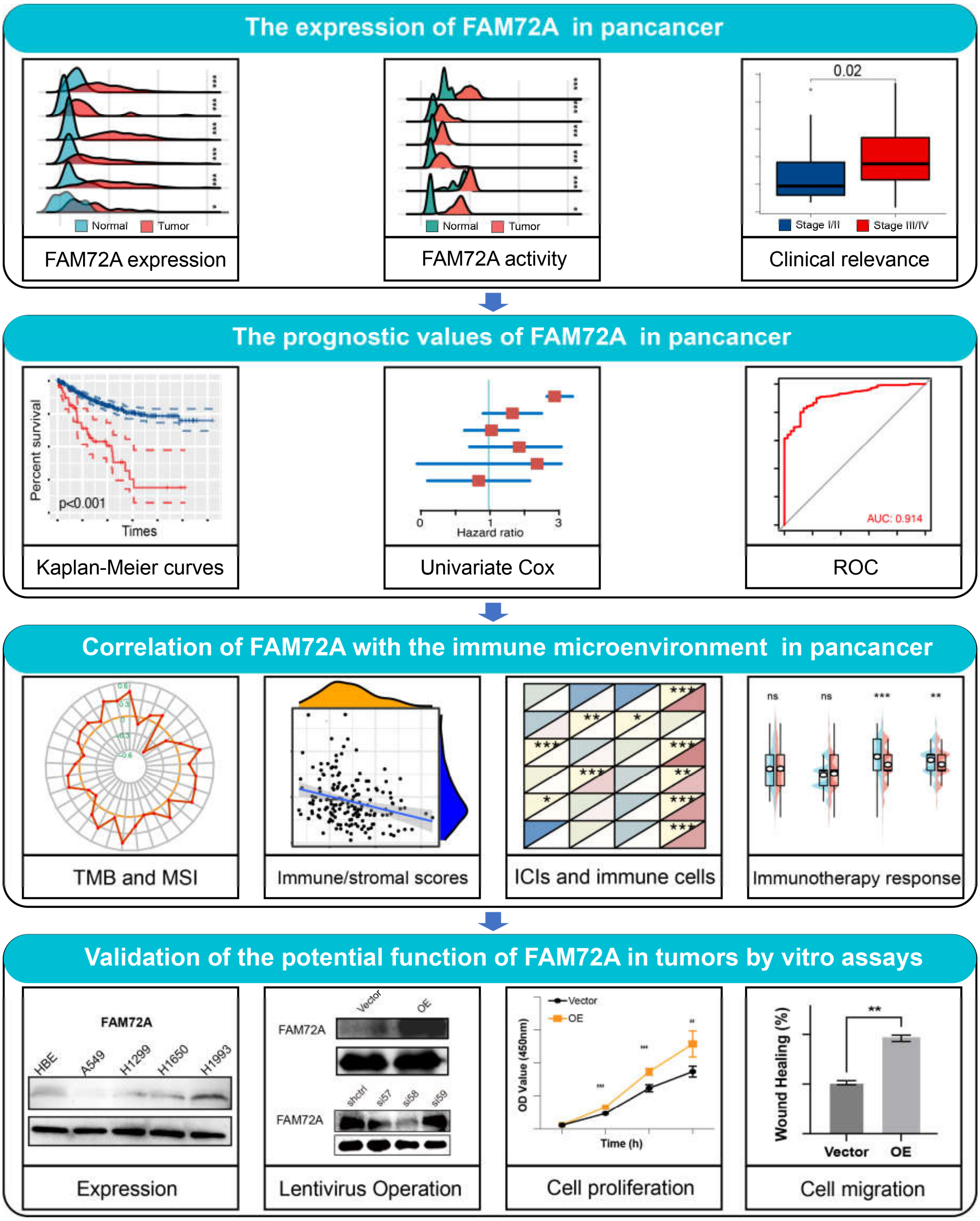

Supplemental Figure S1. The flowchart of the study.

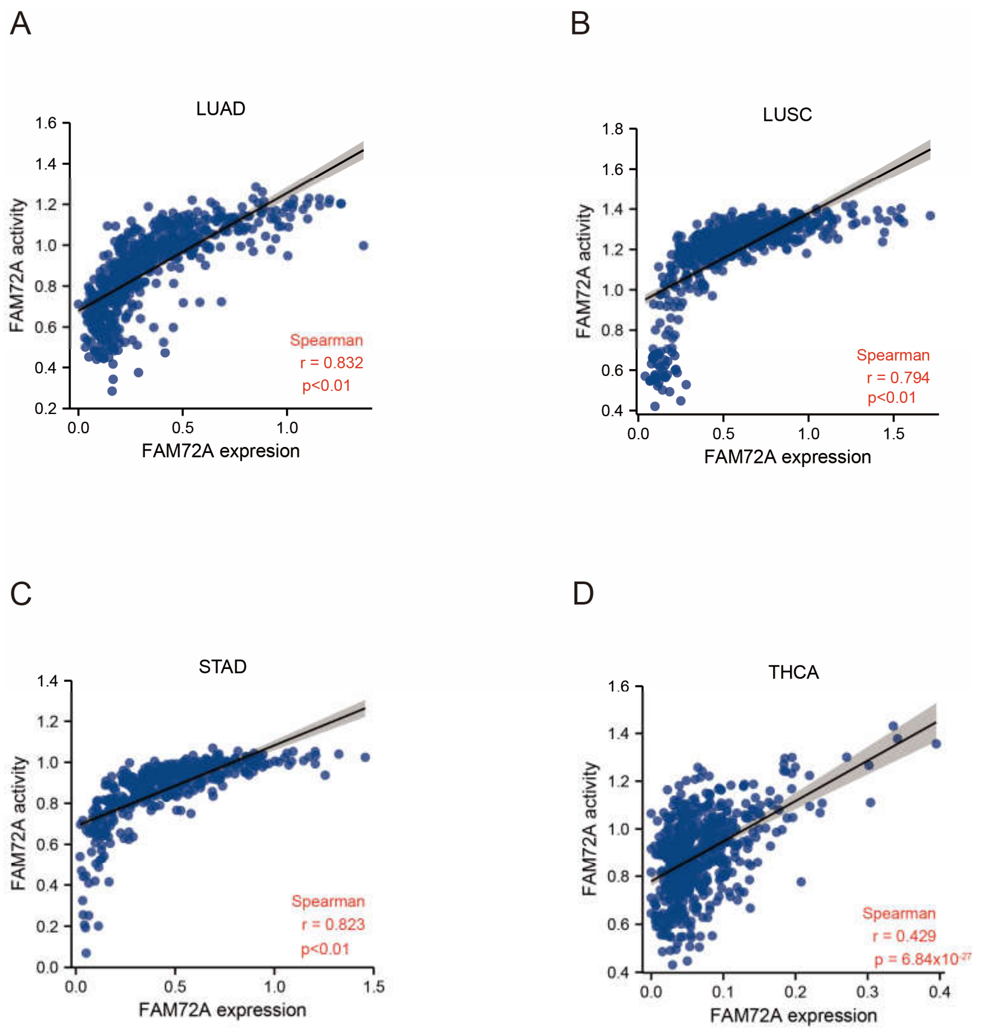

Supplemental Figure S2. Correlation between the expression and the activity of FAM72A in 4 different types of tumors. A-D. Scatter plots for correlation between expression and the activity of FAM72A in LUAD (A), LUSC (B), STAD (C), and THCA (D).

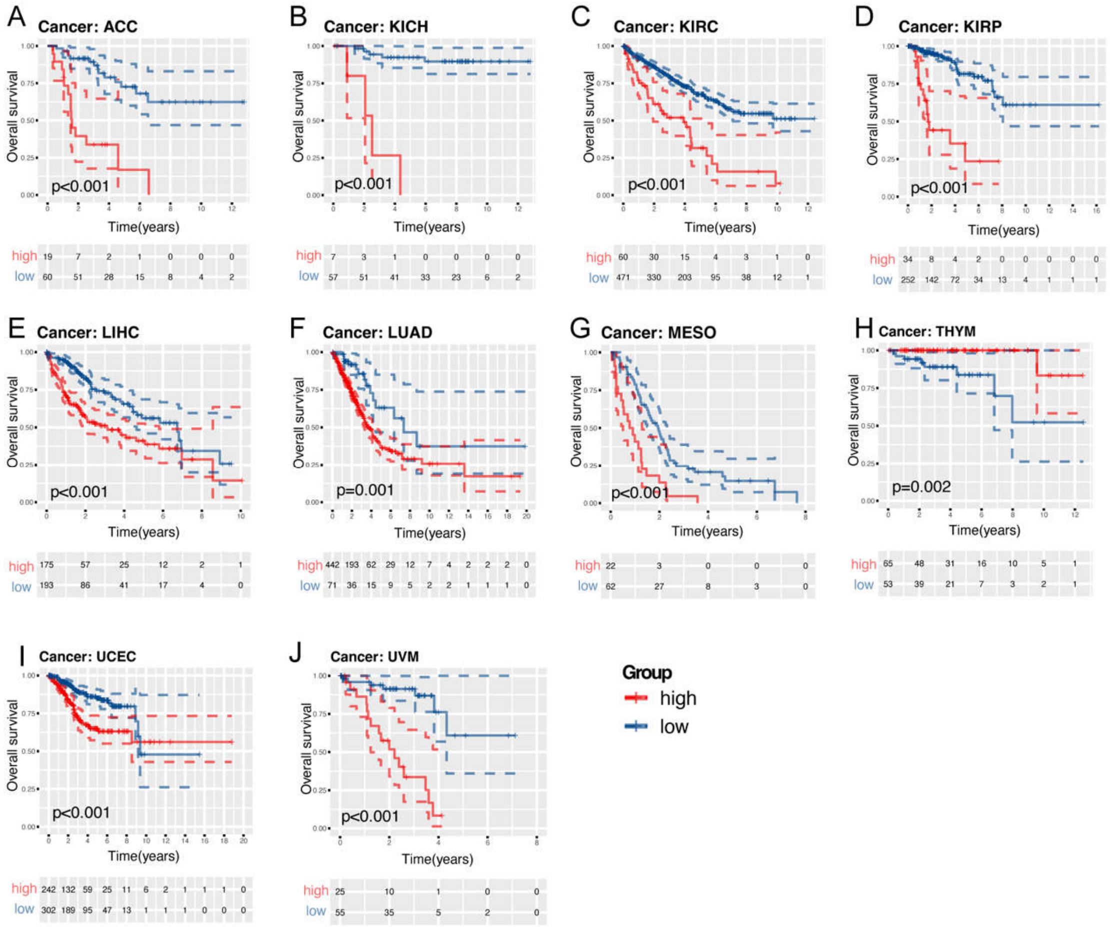

Supplemental Figure S3. Kaplan-Meier survival analysis for the association between the expression of FAM72A and overall survival (OS) of 33 tumors in the TCGA database. A-K. Kaplan-Meier curves for FAM72A^{low} and FAM72A^{high} groups in ACC (A), KICH (B), KIRC (C), KIRP (D), LIHC (E), LUAD (F), MESO (G), THYM (H), UCEC (I), and UVM (J).

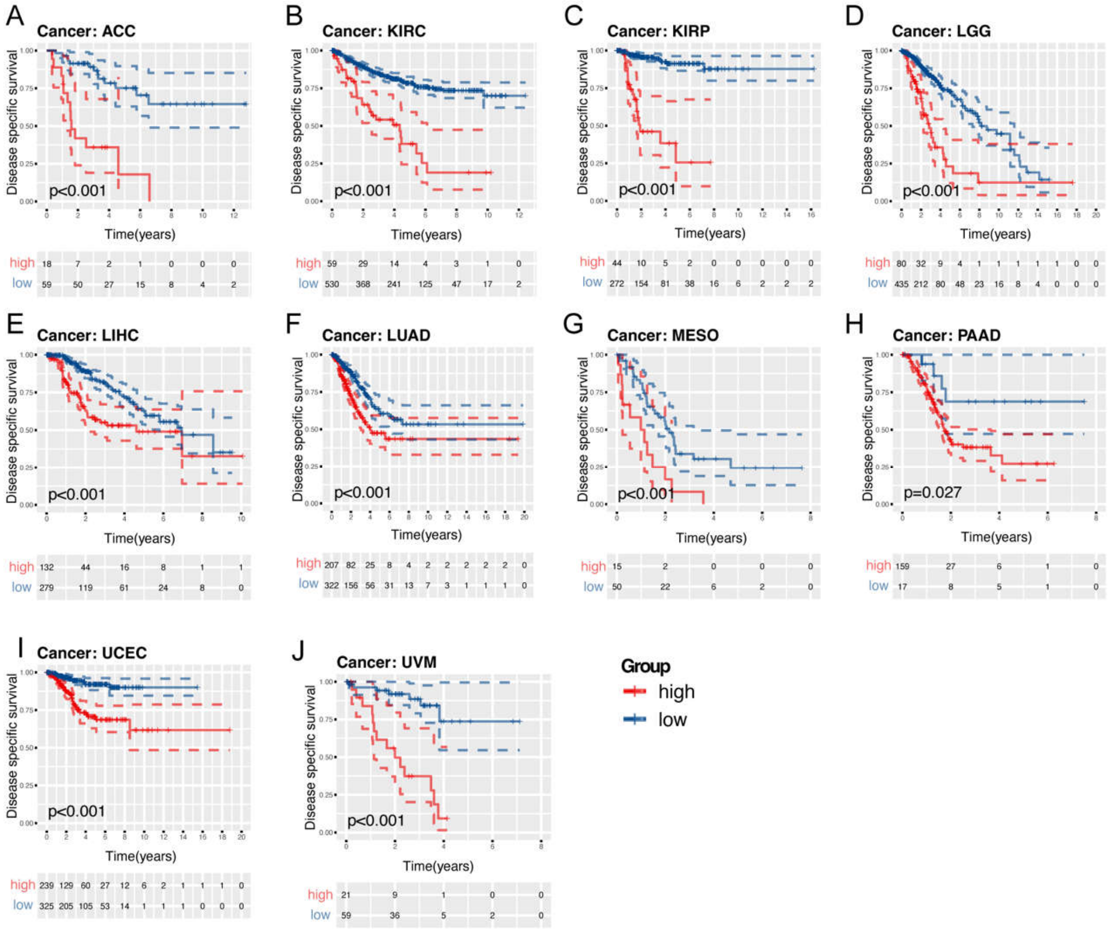

Supplemental Figure S4. Association between the expression of FAM72A and disease-specific survival (DSS) of 33 different types of tumors in TCGA database. A-J. The significant association between FAM72A and DSS of ACC (A), KIRC (B), KIRP (C), LGG (D), LIHC (E), LUAD (F), MESO (G), PAAD (H), UCEC (I) and UVM (J).

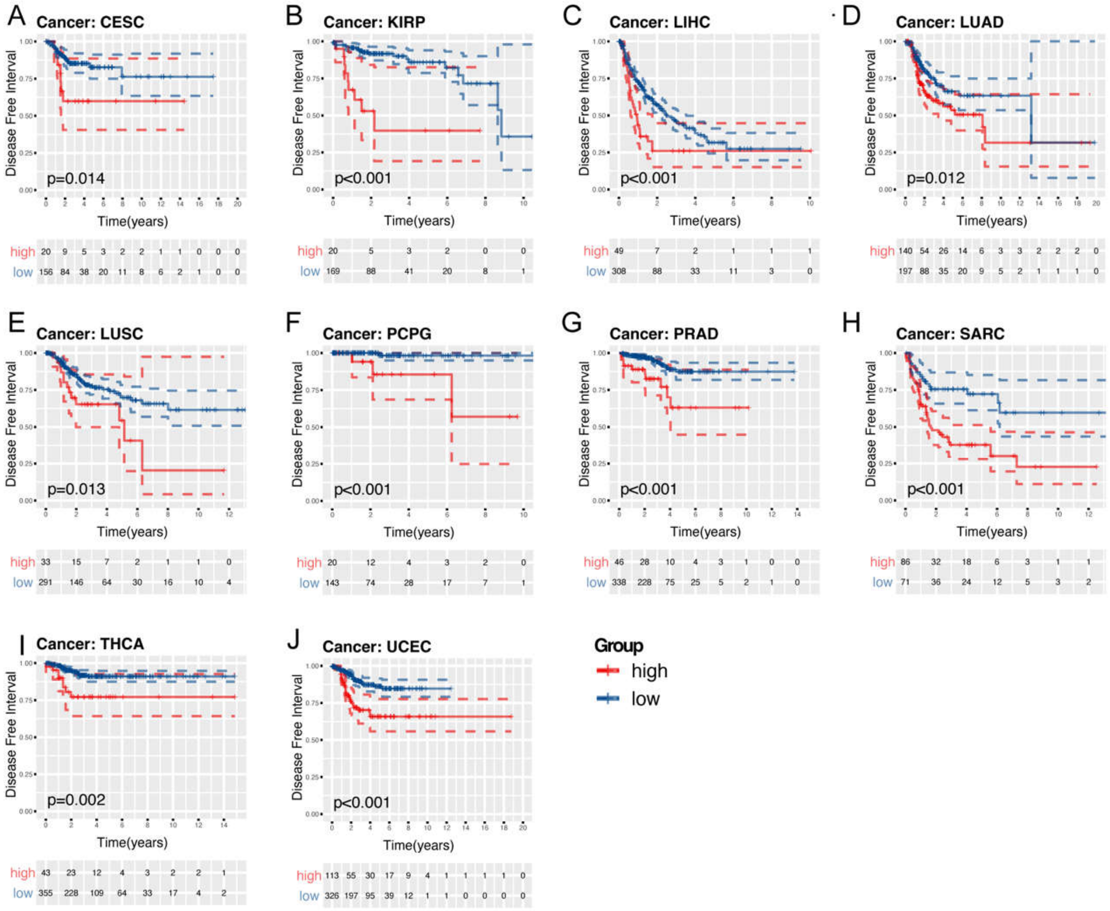

Supplemental Figure S5. Association between the expression of FAM72A and disease-free interval (DFI) of 33 tumors in TCGA database. A-J. The significant association between FAM72A and DFI of CESC (A), KIRP (B), LIHC (C), LUAD (D), LUSC (E), PCPG (F), PRAD (G), SARC (H), THCA (I) and UCEC (J).

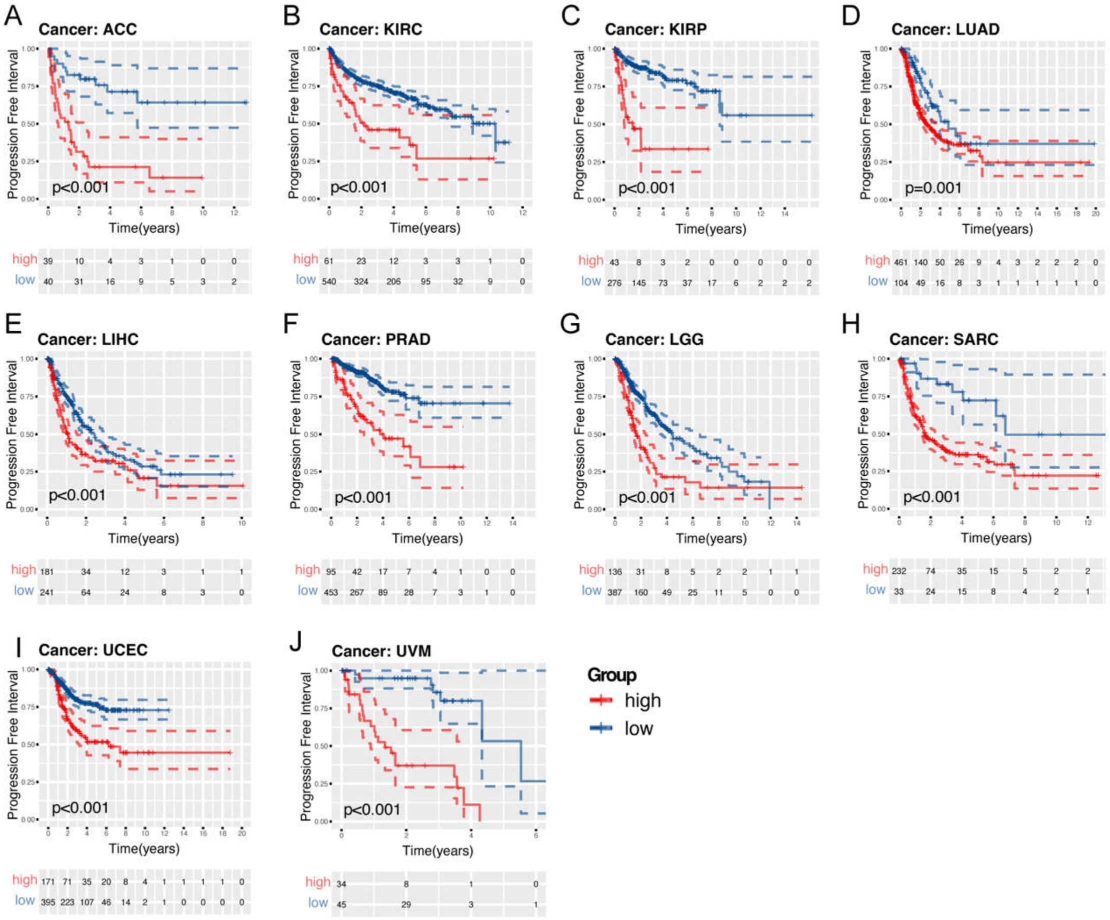

Supplemental Figure S6. Association between the expression of FAM72A and progression-free interval (PFI) of 33 different types of tumors in TCGA database. A-J. The significant association between FAM72A and PFI of ACC (A), KIRC (B), KIRP (C), LUAD (D), LIHC (E), PRAD (F), LGG (G), SARC (H), UCEC (I) and UVM (J).

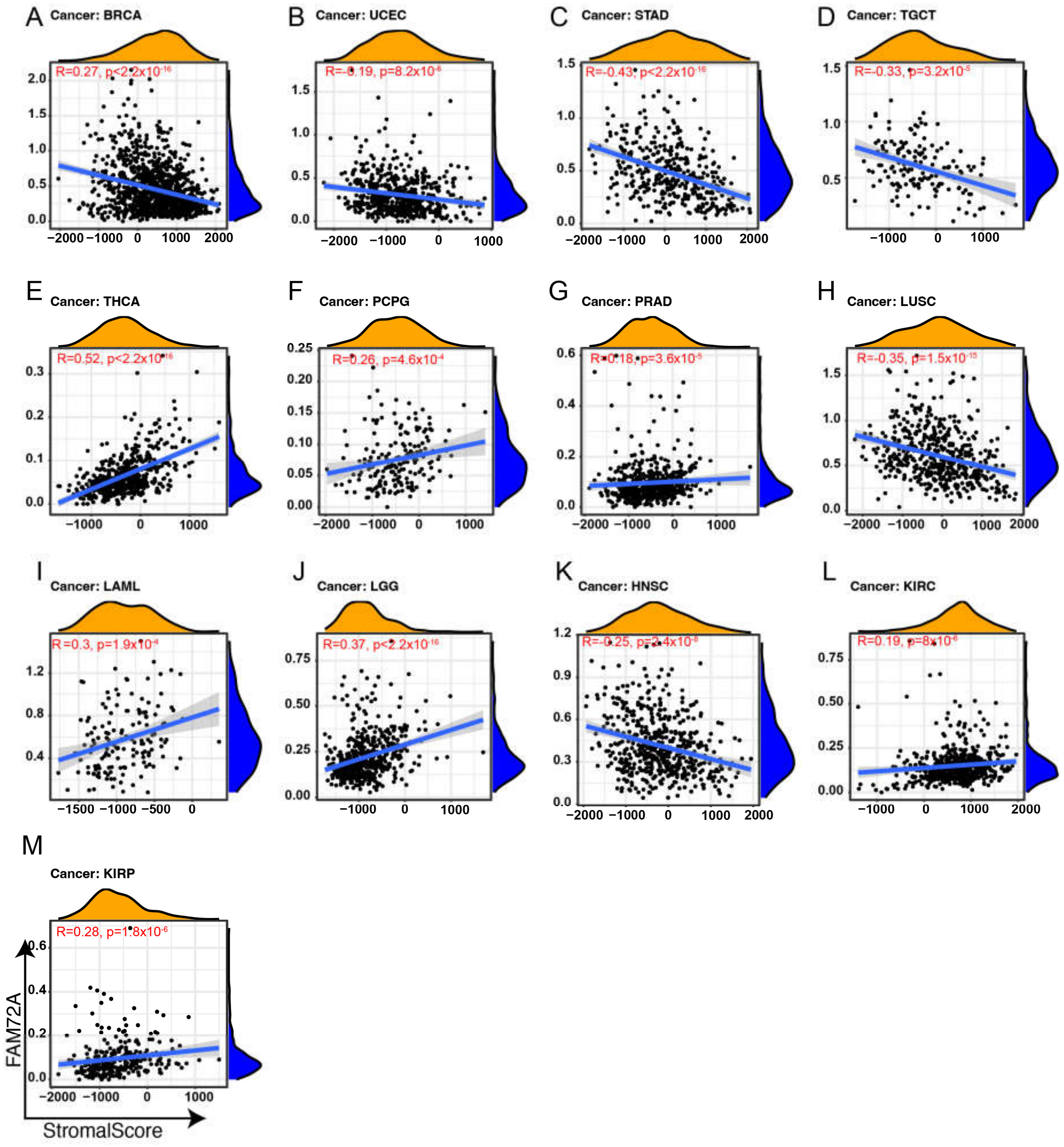

Supplemental Figure S7. Correlation between the expression of FAM72A and stromal scores in pan-cancer. A-M. Correlation between the expression of FAM72A and stromal scores calculated using the CIBERSORT method in pan-cancer. The Spearman method was used to estimate the correlations. ∗p <0.05, ∗∗p <0.01 and ∗∗∗p <0.001
